# Moving Toward an Age-Friendly University: Survey Process of GSU 50+ Students

**DOI:** 10.1093/geroni/igab046.2799

**Published:** 2021-12-17

**Authors:** Grace da Rosa, Jacqueline Laures-Gore, Sarah Barber, Mariam Qureshi, Elisabeth Burgess

**Affiliations:** 1 Georgia State University, Suwanee, Georgia, United States; 2 Georgia State University, Georgia State University, Georgia, United States; 3 Georgia State University, Georgia State University - Atlanta, Georgia, United States

## Abstract

The proportion of Georgia’s population that is 60 years and older is growing rapidly. The 2010 U.S. Census Bureau predicted a growth of more than 20% of older adults by the year 2030. Georgia residents who are 62 and older are eligible to take courses at no or little cost at public state colleges. Due to the expected increase in Georgia’s aging population, access to a free university education, and the large number of currently enrolled 62+ students at Georgia State University (GSU), it is crucial that GSU become an Age-Friendly University. During Fall 2019, a survey was distributed to 1046 students aged 50 years plus; 411 completed the survey (39% response rate). This presentation describes the process involved in designing and distributing the survey. Unique aspects of the survey’s development include the cross-generational and interdisciplinary contributions of the student, faculty, affiliates, and staff from GSU’s Gerontology Institute. The goal was to learn more about GSU students 50 years and older by assessing their motivation for attending school, challenges on campus, perceptions of how the university is currently addressing their needs, factors/resources that have helped/are helping them to adjust to school, and the extent to which they experience age discrimination on campus. The long-term goal of the survey is to use this information to direct GSU in becoming an Age-Friendly University.

